# Exploring the “family-community” co-parenting model to alleviate parental burnout: a case study of Xi’an, China

**DOI:** 10.3389/fpsyg.2025.1646124

**Published:** 2025-09-11

**Authors:** Siyu Li, Baoxin Zhai, Junjia Liu

**Affiliations:** ^1^College of Urban and Environmental Sciences, Northwest University, Xi’an, China; ^2^Shaanxi Xi'an Urban Ecosystem National Observation and Research Station, National Forestry and Grassland Administration, Xi’an, China

**Keywords:** parental burnout, community environment, family-community co-parenting, positive parenting emotions, outdoor parenting activities

## Abstract

**Introduction:**

This study aims to investigate the influence mechanisms of community environments (CE) on parental burnout (PB) among parents of preschool-aged children (0–6 years) in Xi’an, China, while controlling for family environments (FE). A hypothetical framework is proposed to examine the relationships among FE, CE, outdoor parenting activities (outdoor PA), positive parenting emotions (positive PE), and PB.

**Methods:**

Data were collected through an online survey using a professional questionnaire survey platform (Questionnaire Star) from November 2024 to February 2025, resulting in 308 valid responses. Structural equation modeling (SEM) was employed to test the hypothesized relationships within the proposed framework.

**Results:**

The results indicate that CE indirectly alleviates PB by promoting outdoor PA. In contrast, FE plays a more central role in directly reducing PB. The study highlights the effectiveness of a “Family-Community” co-parenting model in mitigating PB.

**Discussion:**

This study provides novel insights by integrating PB and positive PE within the same framework, revealing their multidimensional interactions. The findings offer empirical evidence for developing family policies and community services aimed at alleviating PB in urban settings, emphasizing the importance of considering both family and community factors in interventions.

## 1 Introduction

Parenthood can herald both the rewarding and challenging aspects of human experiences. While it can be profoundly life-affirming and enjoyable, it can also be immensely challenging and frustrating (Mikolajczak et al., 2021). For many parents, raising children is a multifaceted and complex endeavor (Hansen, 2012; Kahneman et al., 2004). When parents chronically lack the necessary resources to manage child-related stressors, they are at heightened risk of experiencing parental burnout, a phenomenon distinct from regular parenting stress (Mikolajczak et al., 2019). Parental burnout (PB) has been defined as “a state of physical and mental exhaustion resulting from double-edged sword of harm on both parents and children, detachment from children, and a diminished sense of parenting fulfillment (Roskam et al., 2017). This stress originates from dynamic interactions within the parent–child relationship, often conceptualized as a mismatch between parental expectations and child behaviors (Abidin, 1983).

The goal of parenting is to cultivate a positive relationship between parents and their children, and to foster their healthy and sustainable physical and mental development. However, parental burnout can undermine this positive parent–child relationship. Numerous studies have demonstrated that parental burnout not only exacerbates parents’ negative emotions and adversely affects their physical and mental health, but also indirectly harms children through maladaptive parenting behaviors (Mikolajczak et al., 2018; Griffith, 2022). Parental burnout creates a vicious cycle of “parental exhaustion → parenting failure → children’s behavioral problems,” which inflicts a double-edged sword of harm on both parents and children. Moreover, it can diminish parental tolerance, resulting in violent reactions to minor provocations and, consequently, child abuse.

Parental burnout is also a significant factor contributing to the decline in fertility intentions. In an empirical study examining low fertility in East Asia, Japanese scholars found that compared to parents with low burnout, those experiencing high parental burnout were 4.2 times more likely to choose not to have additional children (Roskam et al., 2021). This scenario is no exception in China. According to our calculations and analysis of data from the National Bureau of Statistics of China (NBS), the current fertility intention in China has declined to 1.0–1.3 children per family, far below the ideal level of 1.6–1.8 children (National Bureau of Statistics of China, 2024). The total fertility rate (TFR) has fallen to approximately 1.09, well below the replacement level required for population stability (2.1). Even mothers who have given birth to a second child face continued high levels of stress, which reinforces the negative expectations of one-child families regarding multiple parenthood, resulting in few families wanting to have a second child (Krieg, 2007). The post-1990 and post-2000 generations in China tend to practice “intensive parenting,” which demands high-quality child-rearing and thereby exacerbates burnout. If the parental burnout is not effectively addressed, the trend of low fertility may persist. This poses a significant challenge to the demographic structure and social security system. Thus, it is imperative to investigate the factors influencing parental burnout and to develop strategies to alleviate it.

Since the conceptualization of parental burnout in 2018, numerous scholars have systematically evaluated its severity while investigating its contributing factors and underlying mechanisms (Woine et al., 2024; Blanchard et al., 2023; Swit and Breen, 2023; Piotrowski, 2023). The primary determinants of parental burnout can be broadly categorized into two dimensions: family environment and social (community/school) support systems (Roskam and Mikolajczak, 2023). Current research consensus indicates that supportive family and community environments can effectively mitigate parental burnout. Within the family context, key influencing factors include socioeconomic status, intergenerational childcare support and conflicts, parenting ideologies, and family structure (Li et al., 2025). Regarding community environmental influences, early evidence emerged from a 2013 study on immigrant families in Toronto, Canada, which revealed that limited access to community resources and insufficient cultural inclusivity (reduced community friendliness) significantly exacerbated parenting stress among immigrant populations (Chuang and Tamis-LeMonda, 2013). Subsequent research by Markevych et al. demonstrated that exposure to natural environments, such as community parks, could alleviate parental burnout by enhancing the frequency of outdoor parent–child interactions (Markevych et al., 2017). Browning and colleagues further corroborate these findings, suggesting that the long-term benefits of natural environments on child development may indirectly support community safety and the availability of green space, indirectly reducing family parenting stress by promoting outdoor recreation for children (Browning et al., 2022). These recent studies have expanded the understanding about the relationship between community environments and parental burnout, highlighting community environments may serve as protective factors against parental exhaustion.

**Figure 1:**
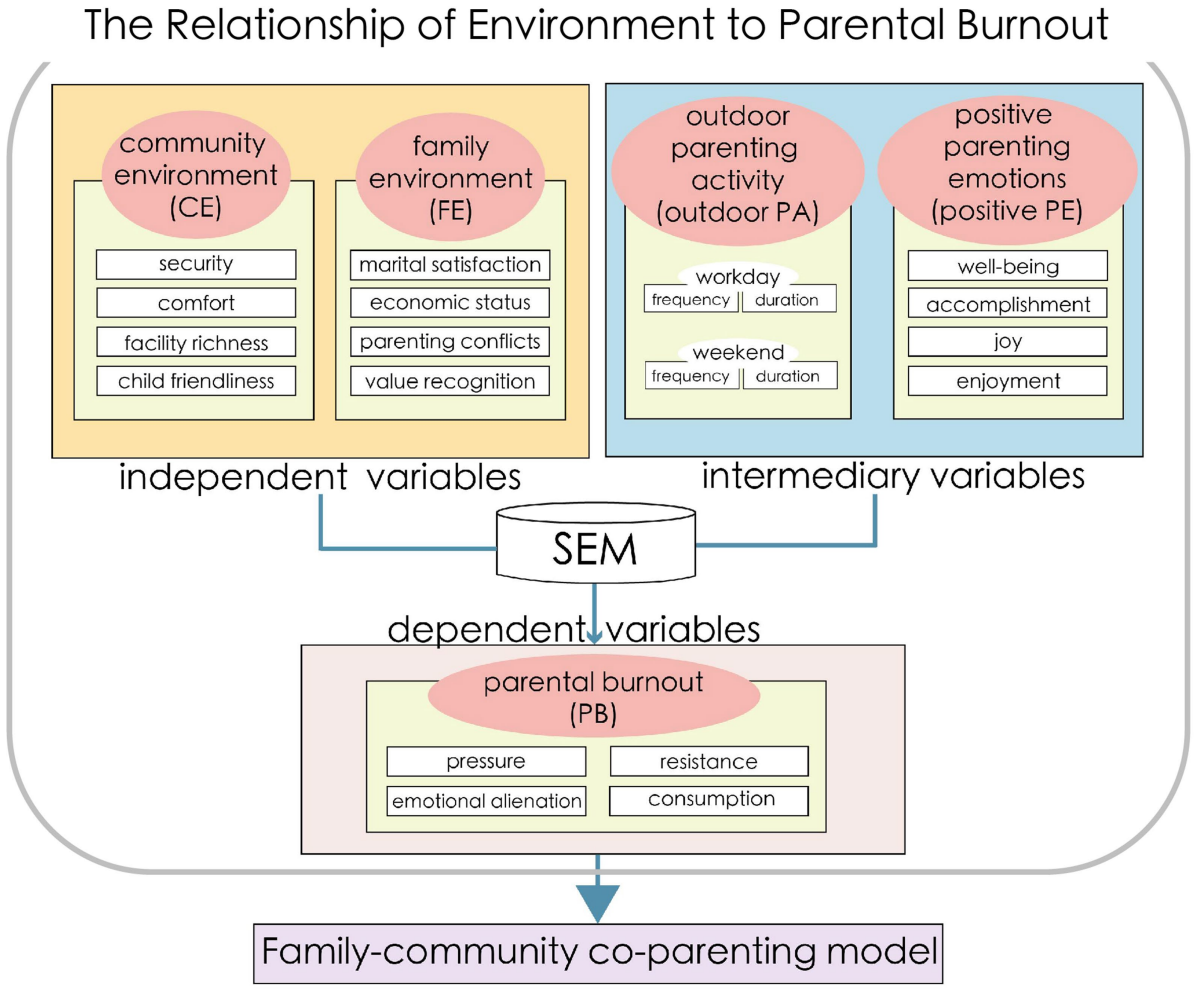
The research framework.

This study aims to elucidate the influence mechanisms of community environments on parental burnout while controlling for family environments. Building on this foundation, we seek to develop a family-community co-parenting model, with a specific focus on alleviating burnout among parents of preschool-aged children. Here, it is necessary to particularly clarify that the co-parenting model mentioned in this paper specifically refers to a mode of joint action that alleviates parental caregiving burnout by improving both the family environment and the community environment, rather than a shared caregiving dynamic model among caregiving responsible individuals. In fact, this model aligns more closely with the foundational framework of Feinberg’s co-parenting model and further elaborates on the role of environmental improvements in the community dimension based on it. The research framework is shown in Figure 1. Drawing on existing research, we first propose a hypothetical pathway examining the relationships among family environment (FE), community environment (CE), outdoor parenting activities (outdoor PA), positive parenting emotions (positive PE), and parental burnout (PB). To test this framework, we will conduct field data collection on PB among families in Xi’an, China, and analyze the hypothesized relationships using structural equation modeling (SEM). By identifying the key factors influencing PB in families with preschool children, this study will support the development of a comprehensive family-community co-parenting system.

## 2 Materials and methods

### 2.1 Research variables

#### 2.1.1 Parental burnout

The PB measures used in this study were adapted from the validated Parental Burnout Assessment (PBA) Scale (Roskam et al., 2018; Cheng, 2020). To ensure conciseness and accuracy, we selected 12 key items from the original scale covering four core dimensions of PB: parenting pressure, resistance to parenting demands, emotional alienation from children, and sense of consumption. The dimensional structure aligns with contemporary theoretical models of PB that emphasize its progressive nature, from initial stress responses to complete emotional withdrawal. However, it is important to note that although the complete scale holds notable advantages in terms of comprehensiveness and detail presentation, the reduction in the number of items in this study does not necessarily imply a substantial weakening of the comparability between this study and international studies that employ the complete scale. In fact, to ensure the scientific rigor and validity of the adapted scale, we conducted a rigorous confirmatory factor analysis on the adapted scale in this study using AMOS software. The analysis results demonstrate that the relevant data fully meet the validity requirements, and detailed validity values can be found in Supplementary Tables S2, S3. This adapted measurement strategy balances methodological rigor with practicality, allowing sensitive detection of burnout severity minimizing respondent burden.

#### 2.1.2 Influence variables

Guided by child-friendly community principles, this study examines the impact of community environments on PB in four dimensions: security, comfort, child-friendliness, and facility richness (Wang, 2023; Hobfoll, 1989; Feinberg, 2003). Security is a fundamental aspect of child-friendly communities, with previous research highlighting parental surveillance convenience (Veitch et al., 2006) and traffic safety (Arzoo, 2024) as critical factors. Delving deeper into psychometric properties, Evans explored in his 2006 article the impact of community design (the visibility between residential areas and playgrounds) on parents’ supervisory burden. He also validated relevant measurement tools, finding that direct visual contact with children’s activity areas can significantly reduce parenting stress (Evans, 2006). Meanwhile, the Traffic Safety Scale (e.g., CTSI) has been extensively validated and is suitable for quantifying the impact of community safety on parental burnout (Molnar et al., 2004). So we operationalize this dimension using two indicators: ease of supervising children’s activities (e.g., visibility of play areas from homes) and pedestrian and vehicular security measures (e.g., speed limits, crosswalks). Comfortable environments mitigate parental stress by promoting children’s outdoor engagement. Empirical evidence suggests that green spaces and optimal building density enhance recreational opportunities while reducing parental anxiety (Faber Taylor and Kuo, 2009). Thus, we assess comfort using two indicators: green space ratio and building density. A truly child-friendly community fosters inclusivity and support. Key elements include: childcare services (e.g., availability of nurseries, after-school programs), community activity inclusiveness (e.g., events tailored for children and families), and neighborhood relationships (Garbarino, 2008). As early as 2009, the ASQ-3, as a psychological tool, demonstrated both cost-effectiveness and favorable psychometric properties in samples from childcare services (Filgueiras et al., 2014). Sirgy and others developed a new metric for measuring community well-being based on the concept of how community residents’ perceptions in areas such as community life and neighborhood relationships regarding the impact of community services and conditions on quality of life (QOL) influence their overall views on community well-being, their commitment to the community, and their overall life satisfaction (Sirgy et al., 2010). Well-structured communities with strong social ties are vital for child development (Negussie et al., 2019). Moreover, families with children frequently utilize recreational facilities, playgrounds, and medical services, making their availability a key determinant of parental burden. Thus, we evaluate the richness of these three types of facilities.

Numerous studies have examined the impact of the FE on PB (World Health Organization, 2021; Mikolajczak et al., 2018). It is believed that family economic status, marital satisfaction, intergenerational parenting conflicts, and the parenting value recognition has the most significant influence on PB (Christian et al., 2017). Scientists discovered the Family Stress Model (FSM) a long time ago. It outlines a theoretical process in which stressors applicable to various family environments, such as economic hardship and stress, primarily affect children’s development in the physical, socio-emotional, and cognitive domains through parental psychological distress (Masarik and Conger, 2017). Therefore, this study employs these four indicators to characterize the FE. In the context of traditional Chinese culture, parents are more inclined to express parenting emotions positively. Thus, in conjunction with the PBA scale, we measure positive PE from four dimensions corresponding to the four dimensions of PB: parenting well-being, parenting sense of accomplishment, parenting joy, and parenting enjoyment. In this study, positive PE are not merely the opposite of PB but serve as complementary evidence to provide a fuller picture of Chinese parents’ emotional state in parenting. The detailed indicators for the influencing factors of PB, please refer to Table 1.

**Table 1:** The setting and distribution of variables in this study.

Variables	Indicators		Mean	S.D.	Min	Max
PB	Parenting pressure		2.10	0.92	1	5
	Resistance to parenting demands		2.00	0.91	1	5
	Emotional alienation from child		1.99	0.85	1	5
	Sense of consumption		2.03	0.96	1	5
Positive PE	Parenting well-being		4.18	0.79	1	5
	Parenting sense of accomplishment		4.14	0.93	1	5
	Parenting joy		3.98	1.02	1	5
	Parenting enjoyment		4.02	0.86	1	5
FE	Family economic status		3.68	0.89	1	5
	Marital satisfaction		4.06	0.76	1	5
	Parenting conflicts		3.37	0.97	1	5
	Parenting value recognition		4.11	0.79	1	5
CE	Security	Parental surveillance	3.80	0.85	1	5
		Traffic security	3.71	1.06	1	5
	Comfort	Green space ratio	3.76	0.99	1	5
		Building density	3.60	0.96	1	5
	Facility richness	Recreational facilities	3.63	1.07	1	5
		Playgrounds	3.59	1.09	1	5
		Medical services	3.87	0.94	1	5
	Child friendliness	Childcare services	3.68	1.09	1	5
		Neighborhood relationships	3.87	0.79	1	5
		Activity inclusiveness	3.76	0.97	1	5
Outdoor PA	Activity frequency in workday (times/week)		2.75	2.56	0	21
	Activities duration in workday (hours/week)		1.90	1.62	0	10
	Weekend activity frequency		2.08	1.59	0	15
	Weekend activity duration		3.32	2.02	0	16

### 2.2 Research data

Xi’an is the capital of Shaanxi Province and the only megacity in the northwestern China. According to the seventh national population census, children aged 0–6 years account for approximately 9.5% of the permanent population in Xi’an (National Bureau of Statistics of China (NBS), 2021). Additionally, the added value of the education industry in Xi’an constitutes 5.0% of the city’s GDP, with early childhood education having the second-highest degree of marketization (Xi'an Bureau of Statistics, 2023). Overall, Xi’an is characterized by a large group of parents raising 0–6 years old children and a robust market demand for assisted parenting. Therefore, this study selects Xi’an as the survey area to provide recommendations for alleviating PB among parents of 0–6 years old in other cities.

In China, children aged 0–6 years are considered preschoolers, and their upbringing is primarily the responsibility of families and communities. Compared with parents of school-age children, who can rely on schools for some support, parents of preschoolers face greater pressure. Hence, the target population for this study’s survey is parents raising children aged 0–6 years. The survey was conducted using the Questionnaire Star platform, with the online questionnaire^1^ distributed to families with children aged 0–6 years in Xi’an from November 2024 to February 2025. A total of 338 questionnaires were collected, and after removing those with missing values and invalid responses, 308 valid questionnaires remained, resulting in an effective recovery rate of 91.12%.

The data sample is gender-balanced, with 49.1% male and 50.9% female respondents. Nuclear families dominate the sample, comprising 74.4% of couples with children and 26.6% of three-generation households. The age distribution of the children is relatively even, covering all age groups from 0 to 6 years. The proportions of children aged 0–1 year, 1–2 years, 2–3 years, 3–4 years, 4–5 years, and 5–6 years are 15.9, 10.4, 14.9, 13.6, 21.8, and 23.4%, respectively. The respondents’ residences are concentrated in the central urban area of Xi’an (see Figure 1). Additionally, the distribution of variables in the data sample is presented in Table 1.

### 2.3 Research method

#### 2.3.1 Structure equation model (SEM)

The purpose of this study was to explore the influence mechanisms of FE and CE on PB, while adding two new factors to explore, outdoor PA and positive PE. Guided by previous research, the following hypotheses were proposed regarding the influence pathways of these five factors:

*H1*: The CE has a direct negative impact on PB.

*H2*: The CE has a direct positive impact on positive PE.

That is, the better the CE, the lower the PB and the higher positive PE.

*H3*: Outdoor PA have a negative impact on PB.

*H4*: Positive PE can alleviate PB.

*H5*: The CE has a direct positive impact on outdoor PA.

*H6*: The FE has a direct impact on PB.

*H7*: The FE has a direct impact on positive PE.

**Figure 2:**
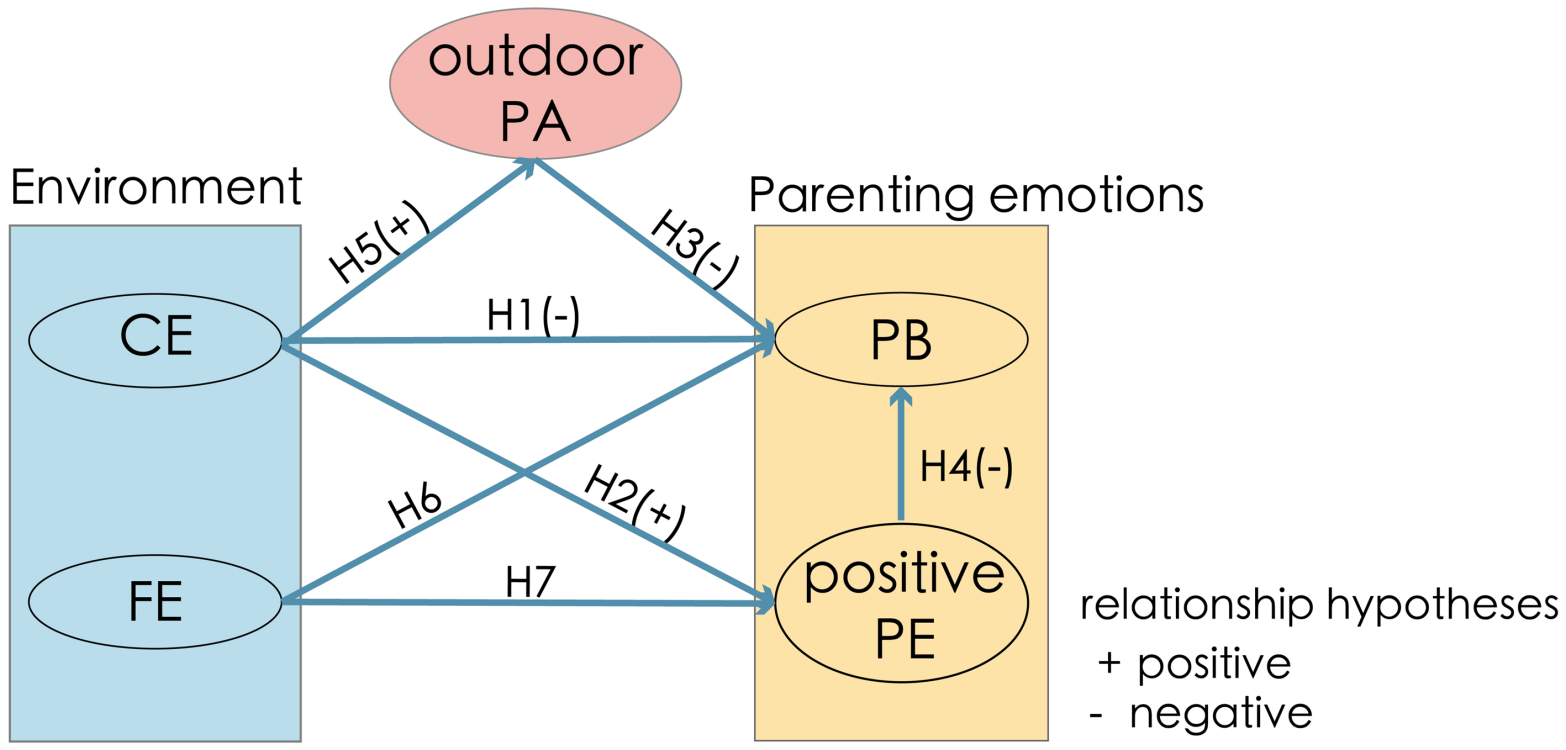
Model assumptions diagram.

Thus, the theoretical hypothesis of the influence paths among PB, CE, FE, positive PE, and outdoor PA is shown in Figure 2. These hypotheses suggest that the CE and the FE also indirectly affect PB by influencing parents’ outdoor PA and positive PE.

Structural equation modeling (SEM) is a commonly used model method for testing and evaluating the fit between the theoretical relationship model and the observed data (Bollen, 1989). Therefore, this study employed SEM to verify the relational hypotheses proposed in Figure 1. In the model, these five factors were treated as latent variables, and the settings of their observed variables were shown in Table 1. The Mplus software is used for the fitting and analysis of the SEM model results. To ensure the accuracy and fit of the model, this study used the Weighted Least Squares Mean and Variance Adjusted Estimation (WLSMV) method for categorical variables and evaluated the model’s fit using multiple indicators. Specifically, this study used chi square test (*χ* ^2^) to evaluate the overall model fit. In addition, approximate fitting indices including RMSEA and SRMR were used to quantify the average magnitude of standardized residual differences. We also prioritized the Comparative Fit Index (CFI) and Tucker Lewis Index (TLI).

#### 2.3.2 Reliability test

The reliability test was utilized to evaluate the rationality of the latent variables and their observed variables in the structural equation model hypothesized in this study. The reliability test was evaluated using two indicators: Composite Reliability (CR) and Average Variance Extracted (AVE). Generally, a CR value exceeding 0.7 and an AVE value above 0.5 are indicative of satisfactory consistency among the items of the measurement variables. The validity test primarily examined discriminant validity between variables, verifying whether the observed variables genuinely reflect the latent variables. It ensures that there is no overlap in measurement between different latent variables (discriminant validity) and avoids conceptual confounds. According to the criteria of Fornell and Larcker (1981), if the correlation coefficient of a variable with others is less than the square root of the average variance extracted from that variable, it has good discriminant validity (Fornell and Larcker, 1981).

## 3 Results

### 3.1 Characteristics

#### 3.1.1 The status of parental burnout

**Figure 3:**
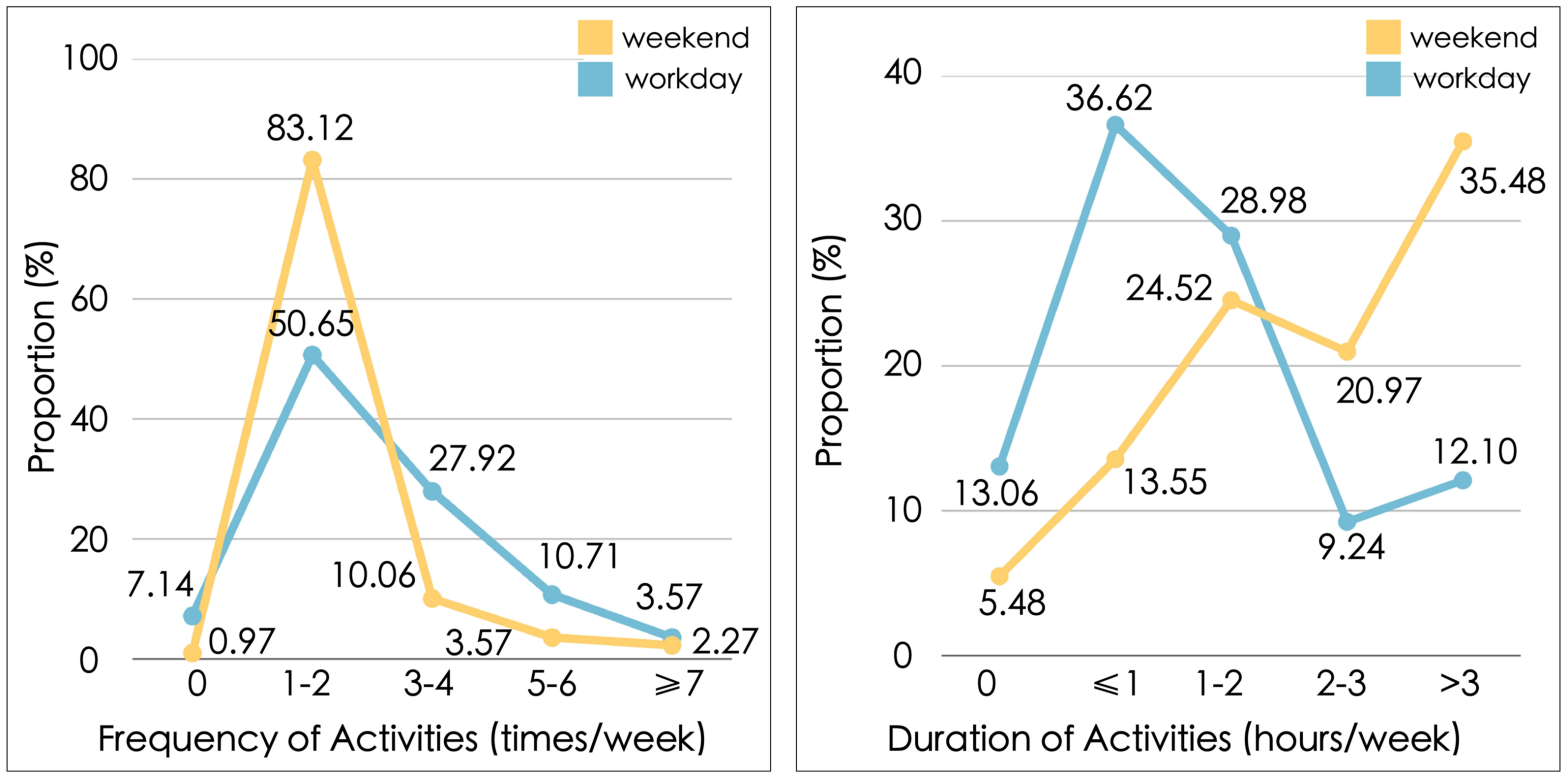
**(A)** Levels of parenting burnout dimensions; **(B)** Levels of positive PE dimensions.

The state of PB and the positive PE among families with preschool children is depicted in Figure 3. Approximately 10.67% of families reported experiencing negative emotions such as parenting stress, resistance, emotional detachment, and depletion. Among these negative emotions, stress was the most prevalent in parenting, with a mean score of 2.14, followed by a sense of depletion (2.06), resistance (2.02), and emotional detachment (1.97). In terms of positive PE, parents generally tend to experience moderate to high levels of positive feelings over the long term in the process of parenting. The strongest feeling experienced in the positive PE was parenting well-being (4.18), followed by sense of accomplishment (4.14), enjoyment (4.02), and joy, which was the least prevalent (3.98).

#### 3.1.2 Characteristics of outdoor PA

**Figure 4:**
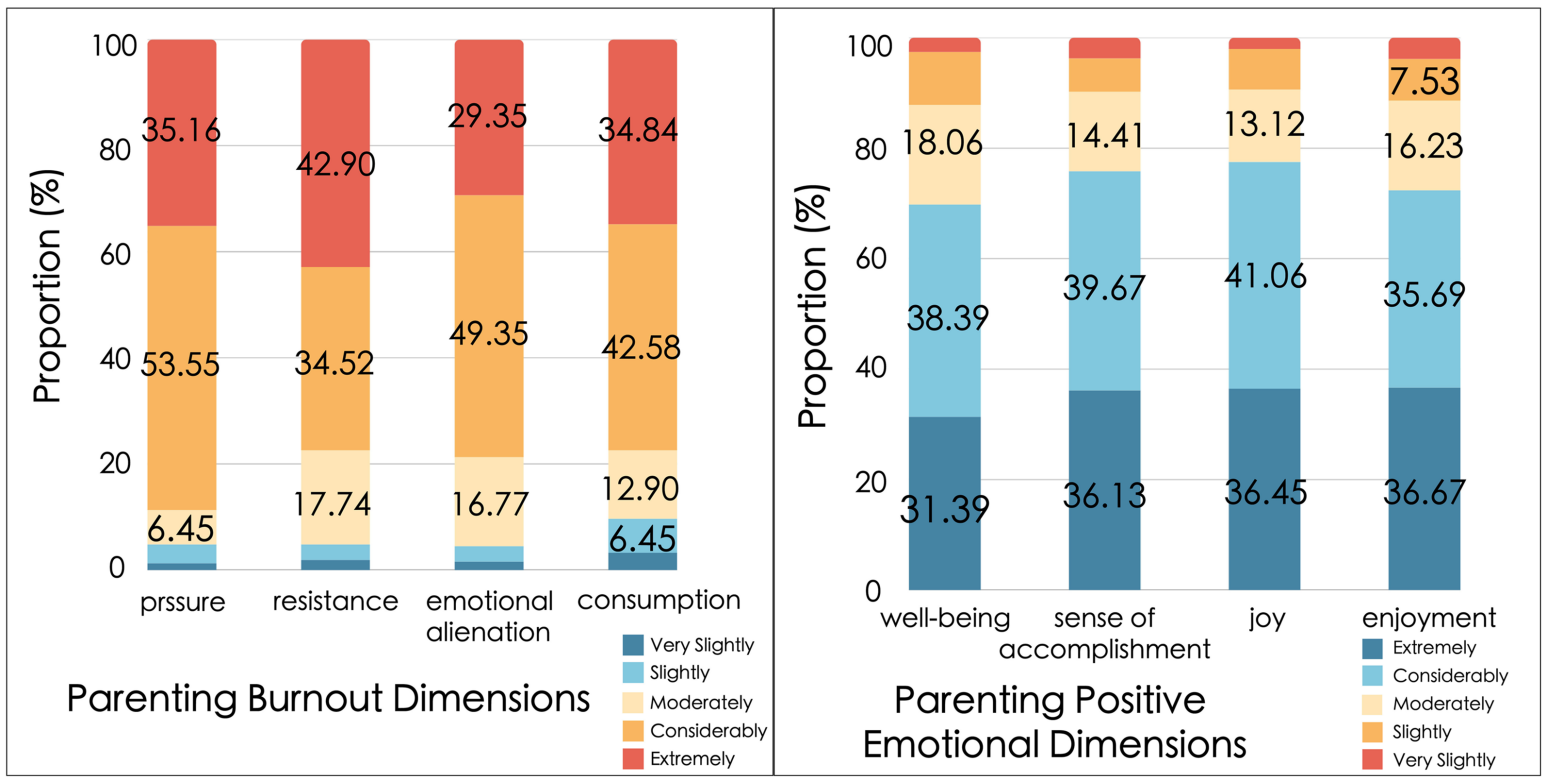
**(A)** The frequency of outdoor PA in workday and weekend; **(B)** The duration of outdoor PA in workday and weekend.

The frequency and duration of outdoor PA on weekdays and weekends are presented in Figure 4. On workdays, 7.1% of parents do not engage in any outdoor PA, while 50.6% participate in such activities 1–2 times per week, and nearly 43% engage in outdoor PA more than three times a week. On weekends, only 0.97% of parents do not engage in any outdoor PA. This indicates that most parents take advantage of outdoor spaces to organize parenting activities. The high frequency of outdoor PA reflects the high demand for outdoor spaces among families with preschool children. Considering the duration of outdoor PA, the average duration per session is 1.90 h on weekdays and 3.32 h on weekends. It can be inferred that the preferred location for most parents’ outdoor PA is likely to be near their residence, i.e., the community where they live, especially during daily. Therefore, the CE is the primary spatial environment that supports parents’ outdoor PA. It revealed that the frequency and duration of parents’ outdoor PA on workday were generally lower than those on weekends.

#### 3.1.3 Correlation between parental burnout and environmental factors

**Figure 5:**
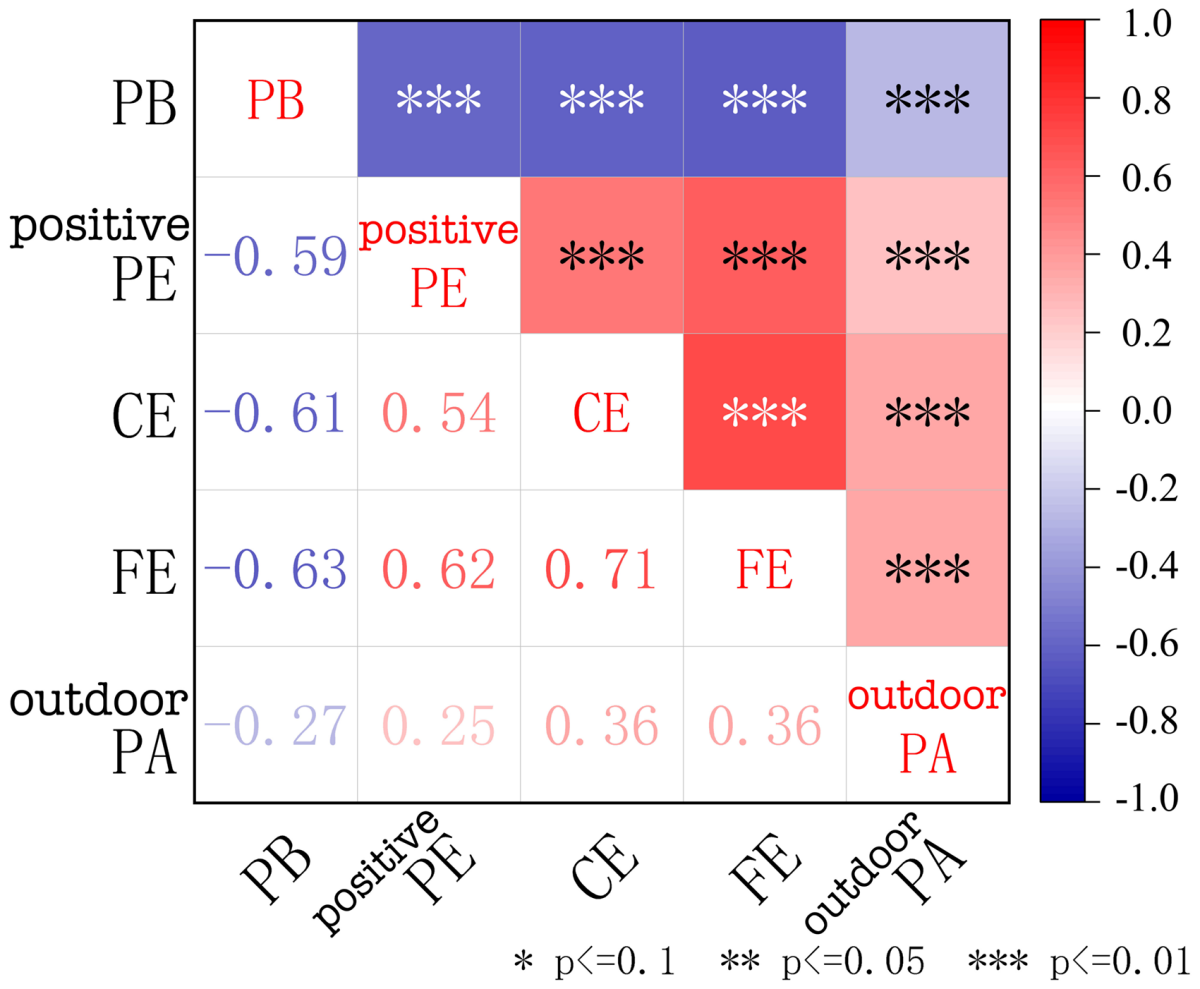
Correlation matrix between parental burnout and environmental factors.

The correlation between the PB, positive PE, and the CE, FE, and outdoor PA are shown in Figure 5. We can find that the CE, FE and outdoor PA show a significant positive correlation with the positive PE and a significant negative correlation with PB. Parents’ positive PE also have a negative correlation with PB. In addition, outdoor PA show a significant positive correlation with the CE. It is plausible that these elements of the CE may also indirectly affect PB by influencing outdoor PA. The conclusions drawn from the above correlation analysis provide some support for our relational hypotheses regarding the impact of the CE on PB. Of course, the relational hypotheses proposed in this study still need further validation through modeling.

**Figure 6:**
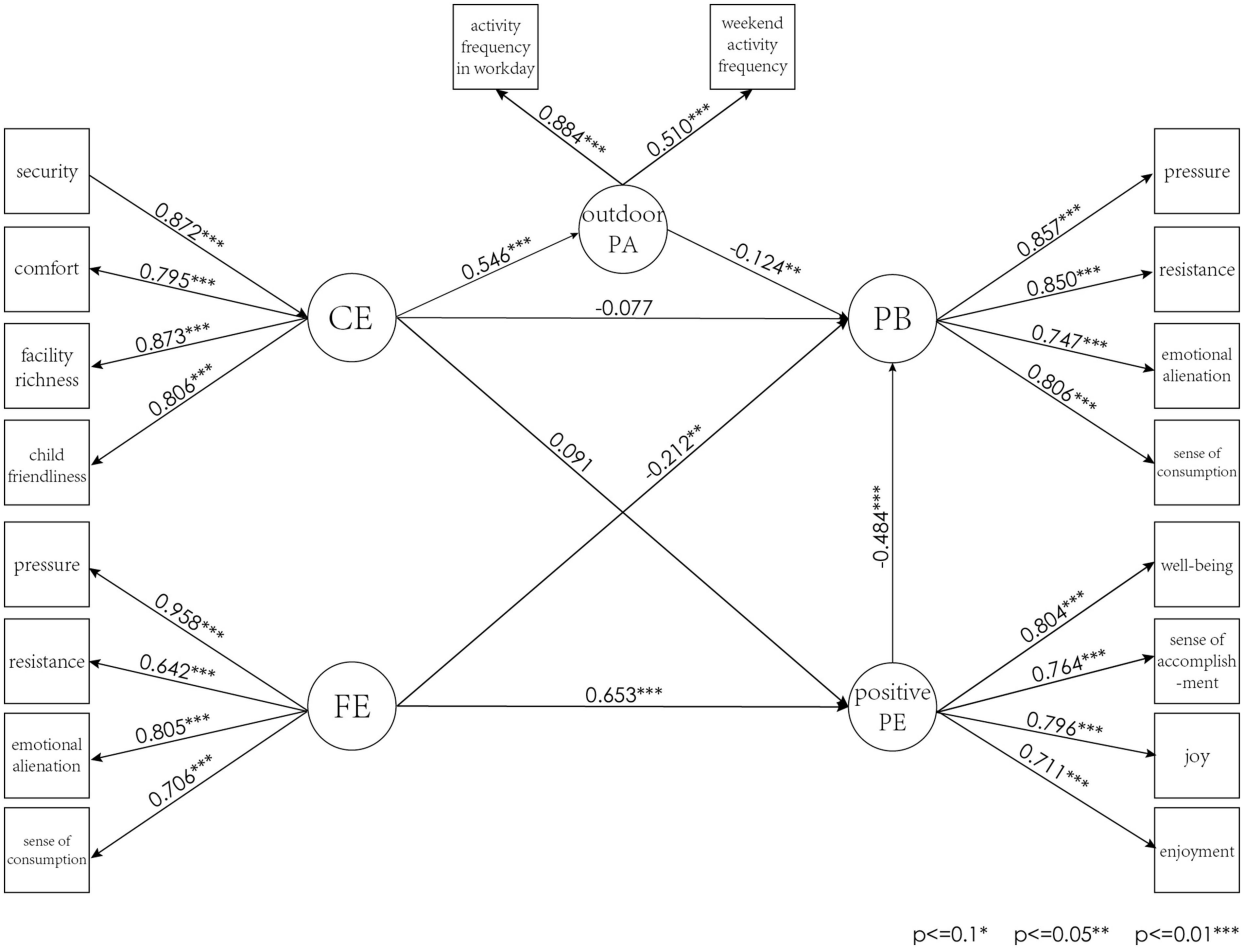
SEM results.

The correlation matrix analysis between specific variables within the research model is shown in Supplementary Figure S1 and Figure 6.

### 3.2 Model results

#### 3.2.1 Model goodness of fit

In the SEM we designed, the results of the CR and AVE values between the five latent variables—PB, positive PE, FE, outdoor PA—and their observed variables are shown in Table S4. The CR values of all latent variables are greater than 0.7, and the AVE values are generally greater than 0.5. This indicates that the internal consistency of the measurement indicators corresponding to each latent variable is good and the reliability is acceptable.

The results of the fit indices for evaluating the SEM model are shown in Table 2. All fit indices of the model are well above the ideal criteria. This indicates that the proposed model is valid. Moreover, the CFI value is 0.975 which further confirms the high fit between the hypothesized model and the empirical survey data.

**Table 2:** Model fit indicators in this study.

Norm	Model indicator values	Standard (Pearson, 1900; Wheaton et al., 1977; Hu and Bentler, 1999)	Model fitness
CMID	282.506*	the smaller the better	
DF	130	the smaller the better	
CMID/DF	2.173	<3 excellent	excellent
CFI	0.975	> 0.95, excellent	excellent
TLI(NNFI)	0.970	> 0.95, excellent	excellent
RMSEA	0.062	< 0.08, good	good

* The chi-square value for MLM, MLMV, MLR, ULSMV, WLSM and WLSMV cannot be used for chi-square difference testing in the regular way. MLM, MLR and WLSM chi-square difference testing is described on the Mplus website. MLMV, WLSMV, and ULSMV difference testing is done using the DIFFTEST option.

#### 3.2.2 Measurement model results

Confirmatory Factor Analysis (CFA) was employed to test the fit of the measurement model and variable relationships. All standardized factor loadings between all latent variables and their observed variables reached statistical significance (*p* < 0.001), and the factor loadings of all observed variables on the corresponding latent variables reached statistical significance (CR > 3.0), indicating that the reliability of the measurement model was high. The results of the measurement model are shown in Table 3.

**Table 3:** Measurement model results.

Observed variables	Relationship direction	Latent variables	Std.	S. E.	C. R.	*p*
Parenting pressure	<−--	PB	0.857	0.032	26.913	0.000
Resistance to parenting demands	<−--		0.850	0.028	30.904	0.000
Emotional alienation from child	<−--		0.747	0.036	21.003	0.000
Sense of consumption	<−--		0.806	0.029	27.411	0.000
Parenting well-being	<−--	positive PE	0.804	0.032	24.882	0.000
Parenting sense of accomplishment	<−--		0.764	0.030	25.110	0.000
Parenting joy	<−--		0.796	0.029	27.233	0.000
Parenting enjoyment	<−--		0.711	0.033	21.442	0.000
Marital satisfaction	<−--	FE	0.958	0.010	93.000	0.000
Parenting conflicts	<−--		0.642	0.039	16.616	0.000
Family economic status	<−--		0.805	0.028	28.677	0.000
Parenting value recognition	<−--		0.706	0.036	19.448	0.000
Security	<−--	CE	0.872	0.025	35.032	0.000
Comfort	<−--		0.795	0.030	26.535	0.000
Facility richness	<−--		0.873	0.022	40.065	0.000
Child friendliness	<−--		0.806	0.027	30.351	0.000
Activity frequency in workday	<−--	outdoor PA	0.884	0.007	118.377	0.000
Weekend activity frequency	<−--		0.510	0.039	13.111	0.000

Pressure, resistance, emotional alienation, and sense of consumption all have positive relationships with PB, with strong standardized factor loadings exceeding 0.7. Specifically, pressure has the highest explanatory value (0.857), followed by resistance to parenting demands (0.850), sense of consumption (0.806), and emotional alienation (0.747). These confirm that PB is characterized by high levels of pressure, resistance to parenting demands, sense of consumption and emotional alienation. For positive PE, parenting well-being had the strongest explanatory power (0.804), followed by joy (0.796), accomplishment (0.764), and enjoyment (0.711).

In terms of FE, marital satisfaction, economic status and parenting value recognition are the key indicators, with high standardized factor loads of 0.958, 0.805 and 0.706, respectively. Parenting conflicts recognition also negatively contributes, though with a lower load of 0.642. Overall, a supportive and positive FE is marked by high marital satisfaction, good economic status, strong parenting value recognition, and low parenting conflicts.

The CE is well-characterized by its four dimensions: security, comfort, facility richness, and child friendliness. All dimensions have strong standardized factor loadings exceeding 0.7, indicating robust measurement and significant contributions to the overall assessment. Specifically, facility richness has the highest explanatory value (0.873), followed by security (0.872), child friendliness (0.806), and comfort (0.795). These high loadings confirm that a good CE is marked by high levels of security, comfort, facility richness, and child friendliness.

In terms of outdoor PA, only the frequency of these activities shows a significant relationship with the overall construct, while activity duration is non-significant, the non-significance results are presented in Table S5. Notably, activity frequency in workday has a higher correlation with the overall structure. This suggests that activity frequency in workday is a key indicator of outdoor PA.

#### 3.2.3 Structural model results

The structural model results, as presented in Table 4. Reveal that all the hypotheses except H1 and H2 are supported. Specifically, the CE does not exert a significant direct impact on PB and positive PE, thereby failing to validate H1 and H2. However, it does have a significant positive effect on outdoor PA (Std. = 0.546, *p* < 0.01). Meanwhile, both positive PE (Std. = −0.484, *p* < 0.01) and outdoor PA (Std. = −0.124, *p* = 0.037) significantly suppressed PB. These findings underscore the mediating role of outdoor PA in the relationship between the CE and PB. It also supports the theoretical hypothesis that outdoor PA act as crucial mediating variables. This finding highlights the need for more comprehensive community designs that not only promote outdoor activities but also provide additional support systems to enhance the overall parenting experience.

**Table 4:** Model path analysis results.

Variables			Std.	S. E.	C. R.	*P*
CE	--->	Outdoor PA	0.546	0.050	11.010	0.000
FE	--->	Positive PE	0.653	0.109	5.995	0.000
CE	--->		0.091	0.110	0.828	0.407
FE	--->	PB	−0.212	0.104	−2.036	0.042
CE	--->		−0.077	0.102	−0.756	0.450
Outdoor PA	--->		−0.124	0.060	−2.088	0.037
Positive PE	--->		−0.484	0.087	−5.574	0.000

The FE also emerged as the most significant factor in enhancing positive PE and reducing PB. It demonstrated a strong positive impact on positive PE (Std. = 0.653, *p* < 0.01) and a substantial negative effect on PB (Std. = −0.212, *p* = 0.042). This suggests that a supportive family system acts as a primary protective factor against burnout. A supportive FE not only provides emotional and practical resources but also fosters a sense of security and well-being, which are crucial for effective parenting.

## 4 Discussion

### 4.1 “Family-community” co-parenting model

The results suggest that the parenting support system needs to form a synergistic structure of “family core strengthening and community scene empowerment,” and it is difficult to alleviate PB by relying solely on the transformation of the physical space. The activation of positive PE and the enhancement of family support quality may be the key to break through the bottleneck.

#### 4.1.1 Family support for parenting

The strong positive effect of the FE on positive PE and its substantial negative effect on burnout underscore the critical role of family support in mitigating burnout. A positive FE provides a foundation for effective parenting by offering emotional, practical, and social support. This support can help parents manage stress, enhance their sense of competence, and foster positive relationships with their children.

This study suggests starting with improving the FE to harmonize parenting emotions. Marital satisfaction, as a core indicator, not only directly enhances the emotional stability of parenting but also strengthens psychological capital through positive feedback loops. When marital satisfaction reaches a certain level, couples can form a stable alliance to coordinate parenting, ensuring that the matter of parenting is not isolated. Simultaneously, addressing the differences in parenting concepts with the older generation and enhancing parenting value recognition can promote more harmonious family relationships and provide spiritual comfort and encouragement for primary caregivers. The interventions aimed at improve family relationship and reduce conceptual conflicts, such as family counseling, parenting workshops, and support groups, can be highly effective in reducing burnout and promoting positive PE. Additionally, family economic status is also a core predictor of PB. Economic stability significantly increases self-efficacy, reduces survival anxiety, and promotes focused parenting engagement. It is also necessary to develop policies that are beneficial to families (e.g., flexible work schedules, tax incentives, childcare allowances) to strengthen the supply of economic resources for parenting. Such family-friendly policies can further enhance the protective effects of the FE on the PB.

Starting from the smallest unit—the family—an assessment of family functioning should be incorporated into the public service system. A family-friendly policy system also should be established to support family relationship and material life. This is of great significance to alleviate family PB.

#### 4.1.2 Community support for parenting

The lack of a direct impact of the CE on PB and positive PE indicates that community-level factors alone may not be sufficient to address burnout directly. However, the significant positive effects on outdoor PA suggest that the CE can indirectly influence burnout through these mediating variables. This highlights the importance of creating CEs that support positive PE experiences. For instance, community programs that promote social connections, provide parenting resources, and encourage outdoor activities can enhance parents’ emotional well-being and reduce PB risk. The findings suggest that community designs should consider the broader context of parenting support, including community security, environmental comfort, child-friendliness, and facility richness.

The safety dimension builds double protection through physical defense and psychological security. The comfort dimension relies on natural elements and spatial density to regulate physical and mental states. We suggest that the community implement a full speed limit in residential areas and adopt an open group layout to reduce building density, minimize visual dead ends, and construct large green areas. This would facilitate the establishment of a “neighborhood watch” system and enhance the security and comfort of children’s activities through resident patrols and the creation of children’s security maps. The facility richness dimension highlights supporting family parenting with diversified recreational facilities and medical care. For example, enriching recreational and play facilities in the community to encourage outdoor PA and enhance the sense of enjoyment in parenting. The child-friendly dimension relies on institutional design and social capital to realize shared responsibility. Organizing more children’s service activities would enhance community participation, fostering a closer neighborhood and enhancing the sense of well-being, joy, and accomplishment in parenting. Strong neighborhood networks provide instrumental support through collective efficacy, and the design of inclusive activities for children alleviates parenting pressure.

It is imperative to transform the future development of communities into child-friendly environments, which would significantly alleviate the PB in the current era. Establishing Parenting Friendly Community Program would be beneficial to enhance parenting positive experience and suppress PB.

### 4.2 Limitations

This study has constructed a foundational theoretical framework to elucidate the mechanism through which FE and CE influence parenting emotions. We selected the Wenjuanxing platform to conduct our survey among all its registered users. Addressing concerns about potential sample bias, specifically whether the sample might be skewed toward the middle class or high-skilled individuals, it should be noted that the majority of parents of children aged 0–6 are middle-aged, and our sample demonstrates a relatively balanced distribution of annual incomes. Meanwhile, the annual income distribution within the sample is uniform, showing no significant imbalance in terms of technology or income levels. We chose Wenjuanxing as our online questionnaire distribution tool primarily due to its advantage in spatial distribution of survey areas, effectively overcoming the limitation of traditional offline paper questionnaires, which may be concentrated in a few communities. Additionally, as the largest questionnaire survey platform in China currently, Wenjuanxing provides robust support for our data collection efforts. But the study’s conclusions may still have certain limitations in external validity due to its focus on children aged 0–6 in Xi’an City. To overcome this limitation, future research plans to extend the age range of children to 7 years and above and conduct pilot studies in multiple representative cities across China to optimize co-parenting models and promote their broad applicability. By integrating theoretical innovation with practical validation, subsequent research will provide a solid scientific foundation for enhancing family well-being in China and globally. However, it must be emphasized here that, given the certain limitations of this study in terms of sample selection, research environment, and other aspects, when attempting to extrapolate the results of this study to populations with different characteristics or apply them to other scenarios that differ significantly from the research context of this study, it is essential to always maintain a cautious attitude. One should never blindly replicate the findings but must proceed with great care.

At the same time, this study ingeniously incorporates the dimension of parenting positive emotions. Given the distinctiveness of China’s socio - cultural context, the study is a pioneer in utilizing specially–developed measurement tools. While the reliability and validity of these tools have been empirically validated, it’s crucial to acknowledge that, as they are independently developed, they may face limitations in terms of generalizability and comparability with existing, widely–recognized measurement instruments. Furthermore, this study treats PB and positive PE as independent and non-antagonistic constructs, although existing evidence indicates a significant negative correlation between positive parenting feelings and parenting burnout, a bidirectional interactive relationship may exist between the two. Therefore, future research needs to employ longitudinal tracking designs to systematically reveal their reciprocal causation mechanisms and further validate the stability of relevant psychometric properties.

## 5 Conclusion

The purpose of this paper is to examine the influence mechanisms of CE on PB, while exploring a parenting model to alleviate the PB. After controlling positive PE, outdoor PA and FE variables, the paper finds that the CE plays a role in cultivating parenting emotions and indirectly alleviating burnout by fostering outdoor PA. Meanwhile, the FE plays a central role in reducing PB. The implication is that “Family-Community” co-parenting model is an inevitable and effective choice to alleviate PB. Since this study integrates and explores the “family-community” dual-influence model, the results elucidate the mechanisms through which the FE and CE influence parenting emotions via distinct pathways. Notably, this study, for the first time, incorporates both PB and positive PE within the same framework, verifying that they are not mere opposites but rather complex constructs independently influenced by multidimensional factors. It provides a novel perspective for understanding the interactions between PB and CE. This study also offers empirical evidence for the formulation of family policies and the design of community services aimed at alleviating PB in large cities.

## Data Availability

The original contributions presented in the study are included in the article/Supplementary material, further inquiries can be directed to the corresponding author/s.

## Footnotes

^1^ https://www.wjx.cn/vm/YdF4SJq.aspx
