# Supplementary material for: Exploring the “family-community” co-parenting model to alleviate parental burnout: a case study of Xi’an, China
Source: Front Psychol. 2025 Sep 11;16:1646124. doi: 10.3389/fpsyg.2025.1646124 (PMC12461073; doi:10.3389/fpsyg.2025.1646124)
Supplement: Supplementary file 1 [file Data_Sheet_1.pdf]

Supplementary Material

1 Indicators and ways to measure parental burnout

Thematic analysis based on the PBA and the Chinese version of the PBA factor structure yielded 12 key items across four key dimensions. These items reflect the regularity of the internal experience of anxious parents. Items were rated on a 5-point Likert scale.

Table S1: The complete set of measurement parental burnout questions

dimensions	items
parenting pressure	I feel like l can't cope as a parent
	I'm in survival mode in my role as a parent
	I'm ashamed of the parent that l've become
resistance to parenting demands	I feel like l can't take any more as a parent
	I have the impression that I'm not myself any more when I'm interacting with my child(ren)
	I can't stand my role as father/mother any more
emotional alienation from child	I don't enjoy being with my child(ren)
	Outside the usual routines, I'm no longer able to make an effort for my child(ren)
	I'm no longer able to show my child(ren) how much l love
sense of consumption	I find it exhausting just thinking of everything l have to do for my child(ren)
	I feel completely run down by my role as a parent
	I have zero energy for looking after my child(ren)

2 Validity information of the adapted version of the Parental Burnout Assessment (PBA) Scale

The PB measures utilized in this study were adapted from the Parental Burnout Assessment (PBA) Scale, a validated instrument designed to evaluate burnout symptoms among parents.To ensure both conciseness and robust assessment, we systematically selected 12 key items from the original scale.Thus we employed AMOS software to conduct confirmatory factor analysis on the adapted scale developed in this research. The results indicated that the data met the validity requirements, and the detailed validity values are presented in Table S2 and Table S3.

Table S2: Model fit indicators for confirmatory factor analysis

Norm	Model indicator values	Standard [43,44,45]	Model fitness
CMID/DF	2.371	<3, excellent	excellent
CFI	0.963	> 0.95, excellent	excellent
TLI(NNFI)	0.949	> 0.95, excellent	excellent
RMSEA	0.067	< 0.08, good	good
RMR	0.042	<0.05, excellent	excellent

This study conducted an analysis on the four key dimensions of the adapted version of the Parental Burnout Assessment Scale, namely parenting pressure, resistance to parenting demands, emotional alienation from children, and sense of consumption. The results of Composite Reliability (CR) showed that the CR values of the four dimensions ranged from 0.753 to 0.786, all exceeding the acceptable threshold of 0.7. This indicates that the scale offers stable and reliable measurements across these four dimensions. In terms of Average Variance Extracted (AVE), the AVE values of the four dimensions fell between 0.505 and 0.551, all surpassing the commonly used criterion of 0.5. This demonstrates that the scale has good convergent validity across these four dimensions, with observed variables effectively converging on their corresponding latent variables, enabling accurate measurement of the characteristics of parental burnout in each dimension.

Table S3:Results of CR and AVE for the dimensions of the scale

	parenting pressure	resistance to parenting demands	emotional alienation from child	sense of consumption
CR	0.784	0.753	0.773	0.786
AVE	0.548	0.505	0.532	0.551

3 The correlation matrix analysis between specific variables within the research model

The correlation matrix analysis between specific variables within the research model is shown in Figure S1.The results show high internal consistency between each latent variable and its subordinate observed variable.As can be seen from the figure, there is no significant correlation between weekend activity frequency under outdoor PA and emotional alienation and consumption under PB, well-being under positive PE, and family under FE, but there is a significant positive correlation with CE. economic status under FE, but was significantly positively correlated with CE. The relationships among the remaining observed variables were consistent with the correlation between the PB, positive PE, and the CE, FE, and outdoor PA and were significantly correlated.From this we can further hypothesize to explore the mediating path of outdoor PA affecting PB by directly influencing CE.

Figure S1: The correlation matrix analysis between variables within the research model

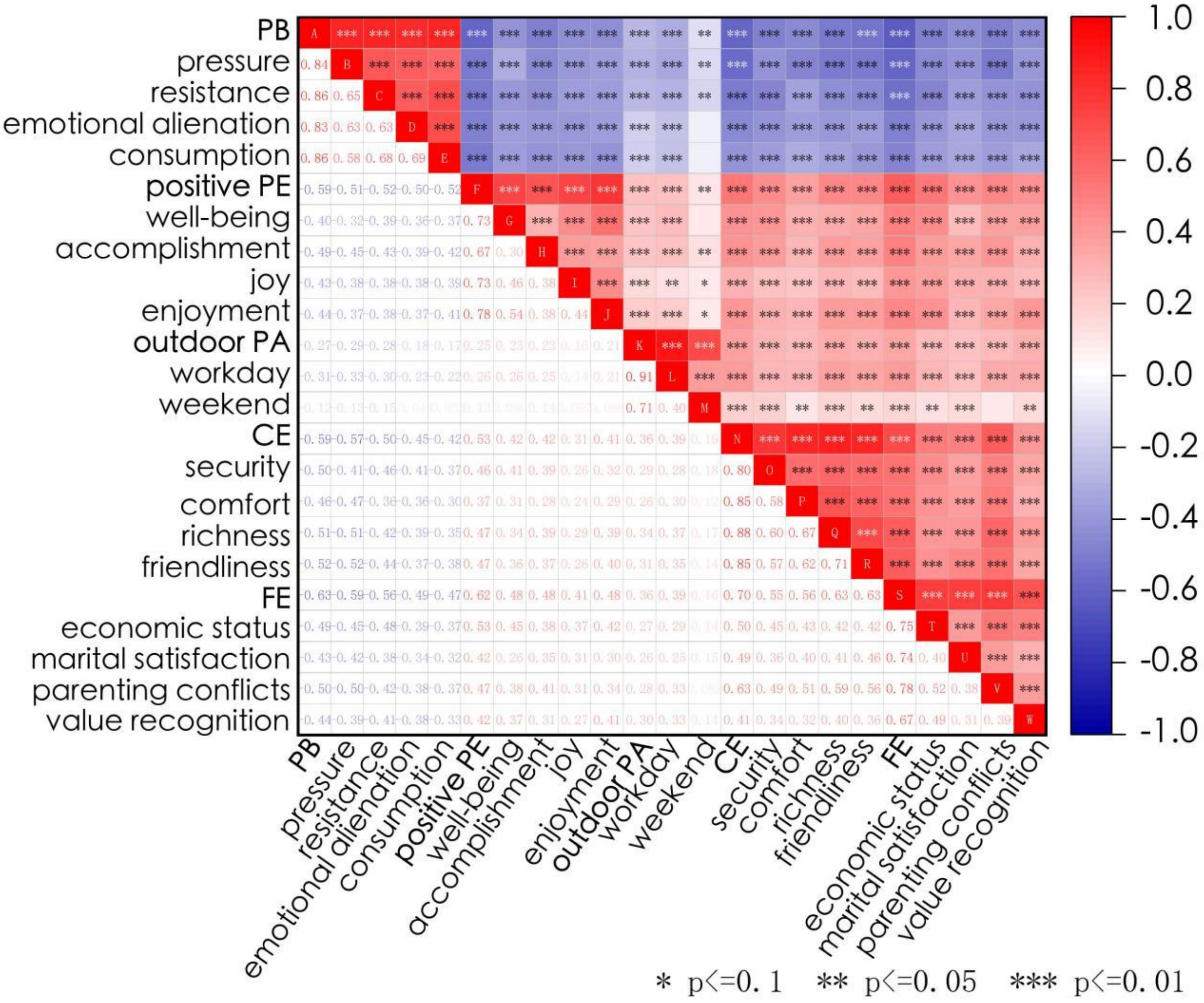

4 Rationality of model variables

In the SEM we designed, the results of the CR and AVE values between the five latent variables—PB, positive PE, FE, outdoor PA—and their observed variables are shown in Table S4. According to the standardized load, SMC, and reliability and validity indicators, except for the weekend activity frequency of outdoor PA (SMC=0.26), the SMC of all indicators exceeded 0.40, and CR and AVE reached the ideal standards (CR>0.80, AVE>0.50), supporting convergent validity. Although the variance explained by weekend activity frequency is relatively low, its theoretical significance as a core indicator of outdoor activity frequency is significant, and therefore it should be retained. Future research can optimize the measurement method of this indicator. This indicates that the internal consistency of the measurement indicators corresponding to each latent variable is good and the reliability is acceptable.

Table S4: Convergent validity checklist

observed variables		Latent variables	SMC	CR	AVE
marital satisfaction	<---	FE	0.918	0.864	0.619
parenting conflicts	<---		0.412		
family economic status	<---		0.648		
parenting value recognition	<---		0.498		
security	<---	CE	0.760	0.903	0.701
comfort	<---		0.632		
facility richness	<---		0.762		
child friendliness	<---		0.650		
activity frequency in workday	<---	outdoor PA	0.781	0.670	0.521
weekend activity frequency	<---		0.260		
parenting stress	<---	PB	0.734	0.888	0.666
resistance to parenting	<---		0.723		

demands					
emotional detachment from child	<---		0.558		
sense of personal depletion	<---		0.650		
parenting well-being	<---		0.646		
parenting sense of accomplishment	<---	positive PE	0.584	0.853	0.593
parenting joy	<---		0.634		
parenting enjoyment	<---		0.506		

5 The rationale for measuring outdoor PA solely based on frequency while disregarding duration

During the process of conducting WLSMV data reanalysis using Mplus software, at the initial stage, we incorporated the frequency and duration data of outdoor activities on weekdays and weekends, which were collected through questionnaires, into the outdoor PA dimension for an overall model analysis. The relevant model significance results are detailed in Table S5. The analysis revealed that the activity duration did not exhibit significance in the current model. Based on this finding, we conducted further in-depth analysis and ultimately decided to remove the activity duration variable from the model, thereby obtaining a model explanation with better fit.

Table S5. Model path analysis results.

Observed variables		Latent variables	Std.	S.E.	C.R.	P
parenting pressure	<---		0.788			
resistance to parenting demands	<---	PB	0.897	0.065	16.891	***
emotional alienation from child	<---		0.796	0.059	14.809	***
sense of consumption	<---		0.769	0.07	14.14	***

parenting well-being	<---		0.774			
parenting sense of accomplishment	<---		0.689	0.088	11.883	***
		positive PE				
parenting joy	<---		0.725	0.081	12.572	***
parenting enjoyment	<---		0.806	0.096	14.014	***
marital satisfaction	<---		0.745	0.105	10.694	***
parenting conflicts	<---		0.57	0.126	8.715	***
		FE				
family economic status	<---		0.716	0.12	10.511	***
parenting value recognition	<---		0.638			
security	<---		0.748	0.07	13.841	***
comfort	<---		0.777			
		CE				
facility richness	<---		0.874	0.071	16.287	***
child friendliness	<---		0.808	0.062	14.966	***
activity frequency in workday	<---		0.888	0.101	4.074	***
activity duration on workdays	<---	outdoor PA	0.008	0.045	0.125	0.9
weekend activity frequency	<---		0.8	0.074	4.074	***
activity duration on weekends	<---		-0.08	0.056	-1.296	0.195

As can be seen from the above tabular data, the path relationships between activity duration on workdays and activity duration on weekends, as well as outdoor PA, are not significant, with p-values of 0.555 and 0.117 respectively. Based on this, we can reasonably explain in the manuscript the rationale for abandoning the principle of "measuring the impact of outdoor activity duration on outdoor parenting activity solely by frequency."
